# Integrated Management of Molar–Incisor Hypomineralisation in a Paediatric Patient: A Case‐Based Perspective

**DOI:** 10.1155/crid/5535412

**Published:** 2026-06-19

**Authors:** Kodikara Mudiyanselage Chathurika Padmakumari, Chandra Godamunne Herath, Hasanthi Sandamala Kumari Ratnatilake

**Affiliations:** ^1^ Department of Oral Medicine and Periodontology, Faculty of Dental Sciences, University of Peradeniya, Peradeniya, Sri Lanka, pdn.ac.lk; ^2^ Department of Community Dental Health, Faculty of Dental Sciences, University of Peradeniya, Peradeniya, Sri Lanka, pdn.ac.lk; ^3^ Department of Community Dental Health, University of Peradeniya, Peradeniya, Sri Lanka, pdn.ac.lk

**Keywords:** caries risk assessment, dental caries, dentistry, first molar management, impacted canine, molar–incisor hypomineralisation, paediatric dentistry

## Abstract

Molar–incisor hypomineralisation (MIH) is a prevalent dental condition that poses significant management challenges. This case report details the multidisciplinary management of an 11‐year‐old female patient diagnosed with MIH, presenting with severe pain associated with the lower right first molar. Clinical examination revealed extensive enamel defects, characterised by demarcated yellowish and whitish hypomineralised areas on her incisors and molars, alongside significant dental caries. A comprehensive treatment plan was developed, involving preventive measures such as oral hygiene education and dietary counseling, alongside restorative interventions. Initial management focused on alleviating pain and restoring function, which included the extraction of nonrestorable teeth and the placement of resin restorations in affected molars. Additionally, the application of fluoride varnishes, and casein phosphopeptide–amorphous calcium phosphate (CPP‐ACP) was implemented to enhance enamel remineralisation. Behavioural management strategies were introduced to address the patient’s dental anxiety, stemming from previous negative experiences with anaesthesia. Follow‐up appointments were scheduled to monitor the effectiveness of treatments and to reinforce oral hygiene practices. The patient’s symptoms improved, root canal and restorative procedures were successfully completed and orthodontic treatment was initiated. She remains under regular follow‐up, demonstrating good cooperation and stable progress. This case emphasises the importance of early diagnosis and a collaborative approach in the management of MIH. By integrating restorative dentistry, preventive care and psychological support, the management plan is aimed not only at addressing the immediate dental concerns but also at improving the patient’s overall quality of life and long‐term dental health.

## 1. Introduction

Molar–incisor hypomineralisation (MIH) is defined as a hypomineralisation of systemic origin that presents as demarcated, qualitative enamel defects affecting one to four first permanent molars (FPMs), often accompanied by involvement of incisors [1]. In 2003, it was further characterised as a developmental enamel defect caused by reduced mineralisation and inorganic enamel components, leading to enamel discolouration and posteruptive enamel breakdown [2]. Although initially described in FPMs and incisors, subsequent reports indicate that any primary or permanent tooth may be affected [3]. Because second primary molars develop during a similar window to FPMs, they too may be involved; this presentation is termed hypomineralised second primary molars (HSPMs) [4].

There is considerable global variation in reported MIH prevalence, ranging from 2.8% to 40.2% across regions and study settings [4]. Using contemporary definitions, it has been estimated that approximately one in six children worldwide may be affected [5]. Much of this heterogeneity reflects differences in study design and diagnostic criteria; to address this, Ghanim et al. developed a structured scoring system aligned with the European Academy of Paediatric Dentistry (EAPD) criteria [4].

The aetiology of MIH remains incompletely understood [6]. Its asymmetrical, demarcated pattern and systemic distribution suggest an insult occurring during the late secretory to early maturation stages of amelogenesis [7]. Proposed contributory factors are likely multifactorial, encompassing prenatal, perinatal and early childhood exposures. These include perinatal complications, respiratory tract infections and episodes of hypoxia, exposure to environmental pollutants such as dioxins, disturbances of calcium–phosphate metabolism, low birth weight, chronic infant illnesses, extended breastfeeding and antibiotic use [6, 7]. A genetic predisposition has also been proposed [8].

Clinically, MIH presents substantial challenges [9]. Affected teeth are often hypersensitive to thermal, chemical and mechanical stimuli, complicating toothbrushing and impairing plaque control, which in turn elevates the risk of caries and gingivitis. Behavioural management can be difficult; previous painful experiences and inadequate anaesthesia may heighten dental anxiety and negatively influence cooperation [5]. These teeth may exhibit chronic pulpal inflammation, contributing to reduced efficacy of local anaesthesia and can be difficult to anaesthetise even with increased doses of local anaesthetic [10]. The treatment burden is high: By age 9, children with MIH are up to 10 times more likely to have received treatment of FPMs than unaffected peers, and affected teeth have often been treated multiple times [5], with additional care frequently required into late adolescence [2]. The condition also carries psychosocial and financial consequences, including impacts on school performance and family resources [4, 9, 11].

For clinical decision‐making, MIH severity is commonly graded as mild, moderate or severe [12]. Optimal care is therefore multidisciplinary, integrating prevention, desensitisation, behaviour guidance, minimally invasive and restorative dentistry and, when indicated, orthodontic planning for strategic extraction of severely compromised FPMs with timely referral. The following case illustrates the coordinated, patient‐centred management of a child with MIH, highlighting assessment, anaesthetic strategies, material selection and long‐term maintenance in the context of the child’s oral and psychosocial needs.

## 2. Case Report

An 11‐year‐old girl presented with her mother to the Division of Paedodontics, Faculty of Dental Sciences, University of Peradeniya, Sri Lanka, complaining of pain in the lower right posterior region of 1 week’s duration. The pain was severe, intermittent and spontaneous, exacerbated at night and had caused several missed school days. A general dental practitioner had attempted management; however, due to poor cooperation, only a temporary restoration was placed on Tooth 46.

Her medical history was significant for multiple hospital admissions within the first 3 years of life. She was born with a birth weight of 2.54 kg and was admitted to the neonatology unit 12 h postpartum for poor sucking. Echocardiography revealed a large atrial septal defect and a large perimembranous ventricular septal defect with moderate pulmonary hypertension, managed with diuretics under paediatric cardiology follow‐up. She subsequently had several admissions for respiratory and urinary tract infections treated with systemic antibiotics. The most recent echocardiogram indicated spontaneous closure of both cardiac defects, with a structurally and functionally normal heart. Prenatal information and maternal illnesses were reviewed, but no relevant factors were identified that contributed to the aetiology of the condition.

She had not attended routine dental care. She is an only child from a low socioeconomic background with limited parental education. Oral hygiene practices were poor: She brushed haphazardly once daily in the morning for about 2 min, without parental supervision, using an adult toothbrush and nonfluoridated toothpaste. Her diet included frequent between‐meal sweets (chocolates, biscuits and sticky toffees) and sweetened drinks. She was breastfed for 4 years and had parafunctional habits of nail, pen and pencil biting.

**Figure 1:**
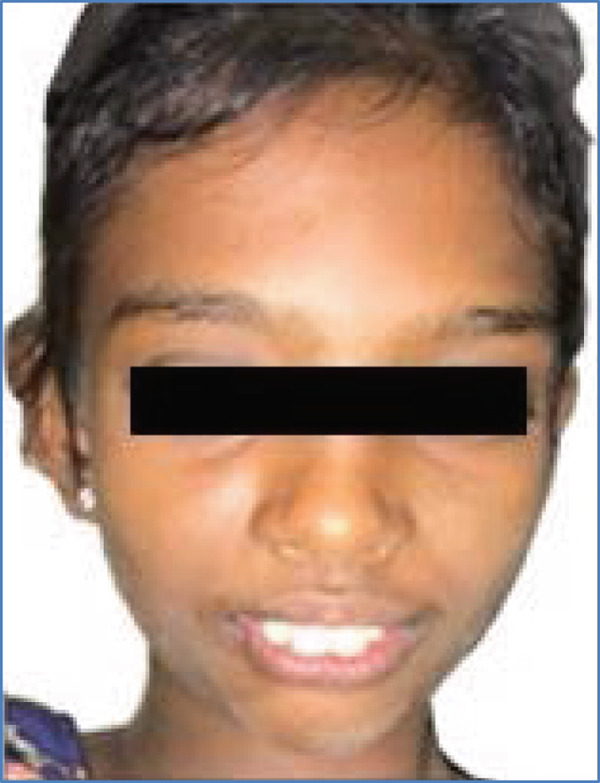
Front facial view.

**Figure 2:**
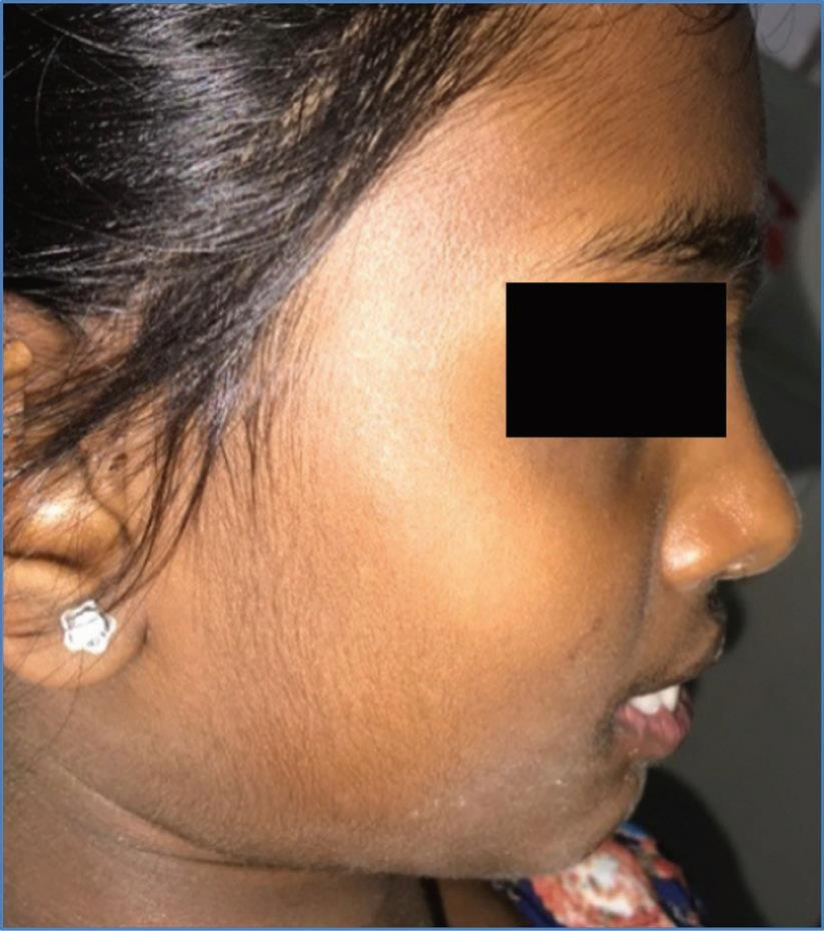
Lateral view.

Extraorally, she had a convex facial profile with a moderate skeletal Class II pattern (Figures 1 and 2), a low Frankfort–mandibular plane angle, everted and grossly incompetent lips and evident lower‐lip trapping. Intraorally, heavy calculus was present around most teeth, with a plaque score of 100%. The gingiva showed physiological pigmentation and mild inflammation.

**Figure 3:**
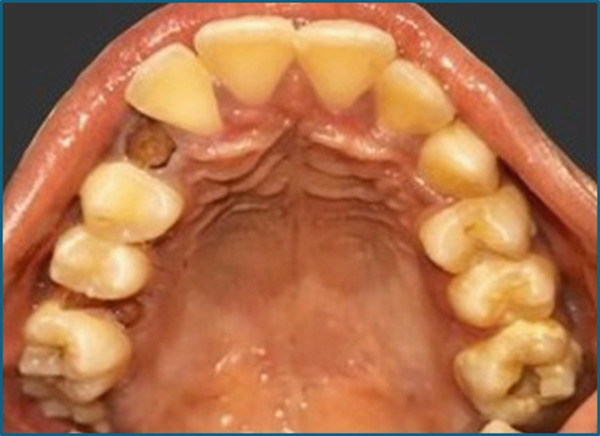
Preoperative upper arch.

**Figure 4:**
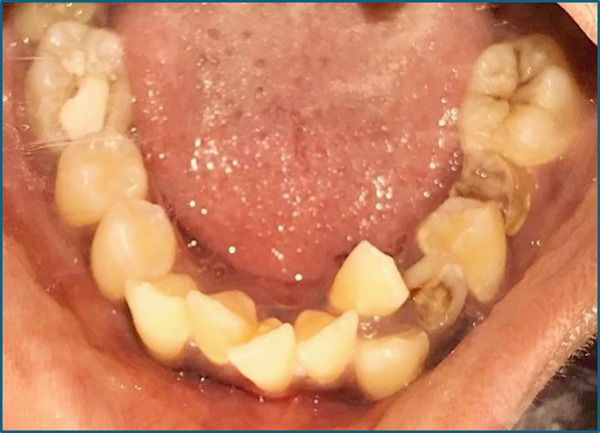
Preoperative lower arch.

**Figure 5:**
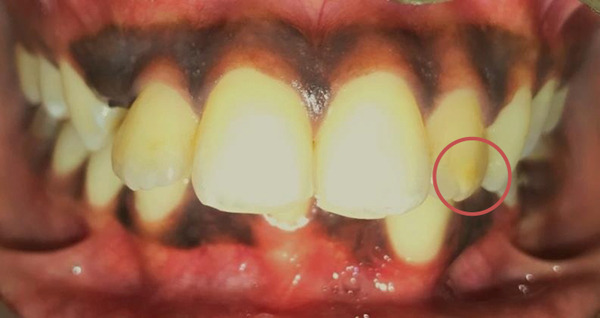
Preoperative front view (hypomineralised patch on 22 is circled).

The permanent teeth present were 11, 12, 14, 15, 16, 17, 21, 22, 23, 24, 25, 26, 31, 32, 33, 34, 36, 37, 41, 42, 43, 44, 45, 46 and 47. Primary Teeth 53, 55, 73 and 75 were grossly carious and overretained. Buccal pit and occlusal caries were noted on 16, 26 and 36; 26 had pulpal involvement (Figures 3 and 4). Tooth 24 had interproximal caries with pulpal involvement. Demarcated yellowish‐white hypomineralised opacities were present labially on 21 and 22, extending up to the incisal third, consistent with MIH (Figure 5). Tooth 46 was temporarily restored. The lower anterior segment was severely crowded with 32 lingually erupted, and 12 was distally angulated.

**Figure 6:**
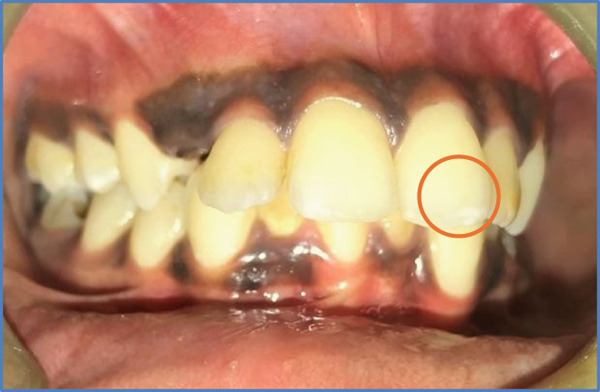
Preoperative left buccal view (hypomineralised patch on 21 is circled).

**Figure 7:**
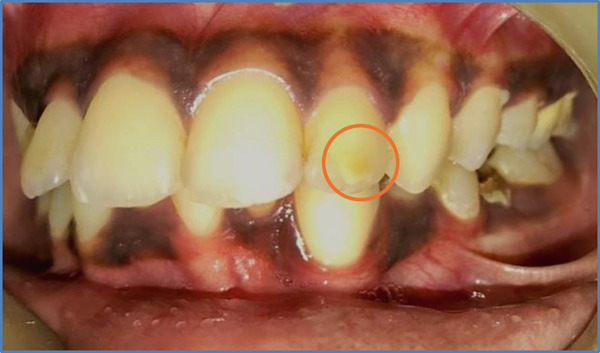
Preoperative right buccal view (hypomineralised patch on 22 is circled).

Occlusal assessment revealed a 9 mm overjet bilaterally with a Class II Division 1 incisor relationship (Figures 6 and 7).

Salivary pH, measured with a commercially available kit, was 6.6 mildly acidic and conducive to demineralisation. A 3‐day diet diary revealed frequent consumption of sugary foods and beverages. Using the AAPD Caries Risk Assessment for patients older than 6 years, the patient was categorised as high risk due to multiple biological and clinical risk factors and few protective factors.

**Figure 8:**
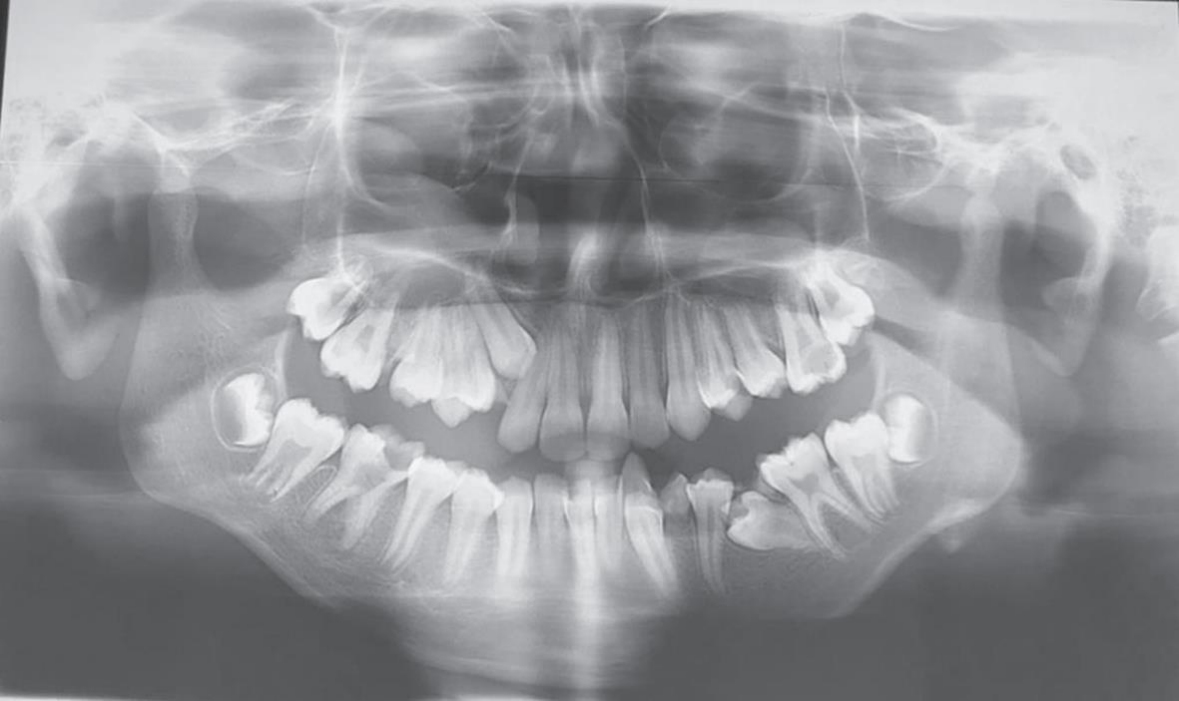
Preoperative dental panaromic tomogram view.

**Figure 9:**
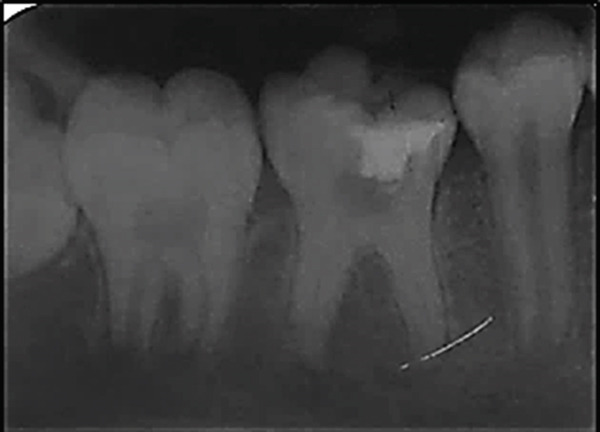
Preoperative radiograph of 46.

**Figure 10:**
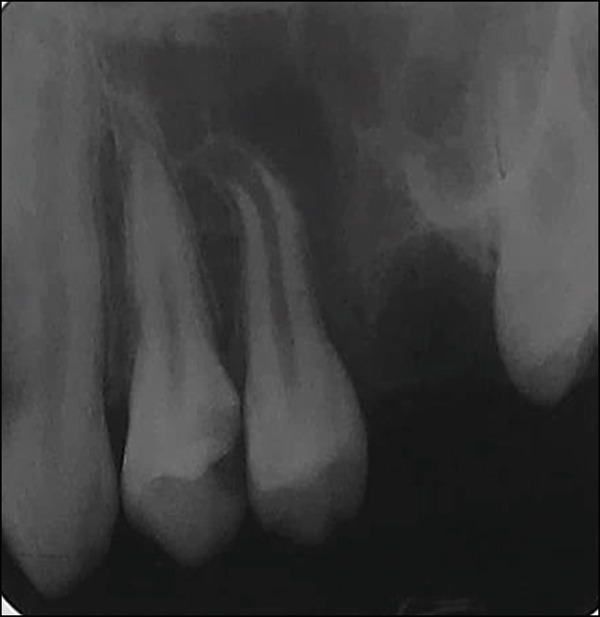
Preoperative periapical radiograph of 24.

The dental panoramic tomogram (Figure 8) showed impaction of Teeth 13 and 35. Tooth 46 exhibited a radiolucency in the coronal dentine extending toward the pulp (Figure 9), with an associated periapical radiolucency and loss of lamina dura at the apices, consistent with pulpal necrosis and periapical pathology. Tooth 26 demonstrated an extensive coronal dentine radiolucency reaching the pulp. Tooth 24 displayed a distal coronal dentine radiolucency approaching the pulp horns (Figure 10). Teeth 16 and 26 showed features of taurodontism (Figure 8).

Based on prognostic factors described by McSherry [13] and Pitt et al. [14], the overall treatment difficulty for realignment of Tooth 13 was assessed as average. The likelihood of eruption of the impacted maxillary canine, according to its position, was as follows:

• Horizontal overlap of the lateral incisor up to half the root width—average prognosis.
• Vertical height of the canine crown from the cemento–enamel junction to less than halfway up the lateral incisor root—good prognosis.
• Canine angulation to the midline of approximately 24°—average prognosis.
• Canine root apex positioned above the first premolar—average prognosis.

Concurrently, Tooth 26 exhibited extensive caries, indicating a long‐term guarded prognosis.

Accordingly, the diagnoses in this 11‐year‐old girl were as follows:

• Generalised mild gingivitis.
• Severe MIH.
• High caries risk (DMFT = 5); reversible pulpitis of 16 and 36; irreversible pulpitis of Teeth 24, 26 and 46; grossly carious, overretained primary Teeth 53, 55, 73 and 75.
• Impacted 13 and 35.
• Class II Division 1 malocclusion with severe crowding in the lower arch.

The following care plan was formulated for the management (Tables 1 and 2).

**Table 1:** Materials used in the present case report.

Material	Commercial name	Manufacturer	City, country
Fluoridated toothpaste	Signal	Unilever	Colombo, Sri Lanka
1% chlorhexidine gluconate gel	Hexigel	ICPA Health Products Ltd.	Ankleshwar, India
			
Daily fluoride mouth rinse	Fluorigard	Colgate‐Palmolive	New York, United States (distributed in Sri Lanka)
5% fluoride varnish	Duraphat	Colgate‐Palmolive	New York, United States (distributed in Sri Lanka)
Casein phosphopeptide–amorphous calcium phosphate (CPP‐ACP)	GC Tooth Mousse	GC Corporation	Tokyo, Japan (distributed in Sri Lanka by local dental suppliers)
Fissure sealant	Helioseal	Ivoclar Vivadent	Schaan, Liechtenstein
Composite restorative material (Tooth 24)	Filtek Z250	3M ESPE	St. Paul, Minnesota, United States
Stainless steel crown (Tooth 46)	Preformed SSC	3M ESPE	St. Paul, Minnesota, United States
Gutta‐percha (root canal filling)	Dentsply Gutta‐Percha Points	Dentsply Sirona	York, Pennsylvania, United States
Root canal sealer	AH Plus	Dentsply Sirona	Konstanz, Germany
Preventive resin restoration material (16, 36)	Filtek Z250	3M ESPE	St. Paul, Minnesota, United States
Resin‐based fissure sealant (premolars)	Helioseal	Ivoclar Vivadent	Schaan, Liechtenstein

**Table 2:** Checklist of procedures described in the present report.

Procedure	Category	Notes/outcome
Pharmacological management for pain and infection (Tooth 46)	Emergency care	Relief of pain and infection
Oral hygiene education, plaque disclosing, brushing demonstration	Preventive care	Patient and parent motivation
Introduction of fluoridated toothpaste, chlorhexidine gel, fluoride mouth rinse	Preventive care	Reinforcement of hygiene
Application of fluoride varnish, CPP‐ACP	Preventive care	Remineralisation support
Full mouth scaling and polishing	Stabilization care	Improved oral hygiene
Extraction of overretained deciduous teeth (53, 55, 73, 75)	Stabilization care	Space management
Root canal treatment of 46 and 24	Definitive care	Pain relief and restoration
Stainless steel crown on 46, composite restoration on 24	Definitive care	Functional rehabilitation
Preventive resin restorations on 16 and 36	Definitive care	Caries control
Extraction of 26	Definitive care	Space for eruption of 27
Orthodontic treatment with Twin Block appliance	Definitive care	Class II correction (interceptive)
Regular fluoride varnish application, monitoring eruption of 27, 13 and 35	Maintenance care	Long‐term follow‐up
Review of hypomineralised patches on 21 and 22	Maintenance care	Conservative monitoring

### 2.1. Emergency Care

• Pharmacological management to alleviate pain and infection in relation to 46

### 2.2. Preventive Care

• Patient and parents’ education regarding the condition and the caries process
• Motivation, guided oral hygiene instructions, brushing demonstration after plaque disclosing
• Introduction of fluoridated toothpastes
• Diet sheet analysis and dietary modification
• Habit intervention for biting nails, pens and pencils
• Introduction of 1% chlorhexidine gluconate gel for 2 weeks, followed by daily fluoride mouth rinse
• Fissure sealants on molars and premolars
• Application of 5% fluoride varnish and repeat it at 3‐month intervals
• Daily use of casein phosphopeptide–amorphous calcium phosphate (CPP‐ACP)
• Oral hygiene reinforcement at each visit

### 2.3. Stabilisation Care

• Full mouth scaling and polishing
• Extraction of overretained deciduous Teeth 53, 55, 73 and 75

### 2.4. Definitive Care

• Root canal treatment for 24 and 46
• Coronal restoration of 24 with composite and 46 with a preformed stainless steel crown (SSC)
• Preventive resin restoration of 16 and 36
• Extraction of 26
• Orthodontic treatment for Class II Division 1 malocclusion

### 2.5. Maintenance Care

• Observation for the eruption of 27 into the 26 position
• Observation for the eruption of 13 and 35
• Three‐monthly reviews for the identification of any new caries development and assessment of maintenance of oral hygiene
• Regular oral prophylaxis (scaling and polishing)
• Three‐monthly application of 5% sodium fluoride varnish
• Review of the hypomineralised patches on incisors—hypersensitivity or any aesthetic concerns
• Regular follow‐up in orthodontic management

On the first visit, the child presented with pain in relation to Tooth 46. Metronidazole 100 mg every 8 h, amoxicillin 125 mg every 8 h and paracetamol 250 mg every 6 h were prescribed to relieve pain and control infection, as she had palpable submandibular lymph nodes and a history of fever prior to presentation. Since the patient was very anxious during the initial visit, invasive procedures were not attempted.

The child and her mother were instructed regarding the carious process and how MIH has been contributed to the risk of the carious process. They were taught about the importance of maintaining appropriate oral hygiene and the requisite of fluoride usage. Then, 1% chlorhexidine gel was recommended for tooth brushing for the first 2 weeks, followed by fluoridated toothpastes. Erythrosine disclosing dye was used for plaque demonstration, and guided oral hygiene instructions were given to the child as well as to her mother. The importance of parental supervision during brushing was emphasised. Fluoride varnish application was done, and a 3‐day diet sheet was given, supplemented with proper instructions.

The diet sheet analysis revealed repeated intake of sugary food and beverages. So, a discussion was conducted with the presence of both the patient and her mother, and possibilities of necessary changes and alternatives to dietary modifications were discussed. After applying different behavioural management strategies, it was possible to obtain the patient’s cooperation for the removal of caries and extirpation of the pulp of 46 under local anaesthesia. It was temporised with glass ionomer cement (GIC), covering all the hypomineralised areas.

**Figure 11:**
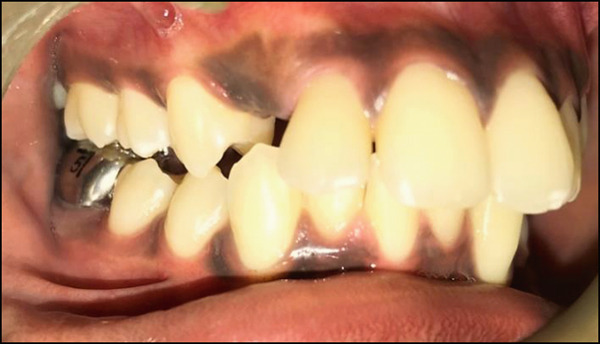
Postoperative right buccal view.

**Figure 12:**
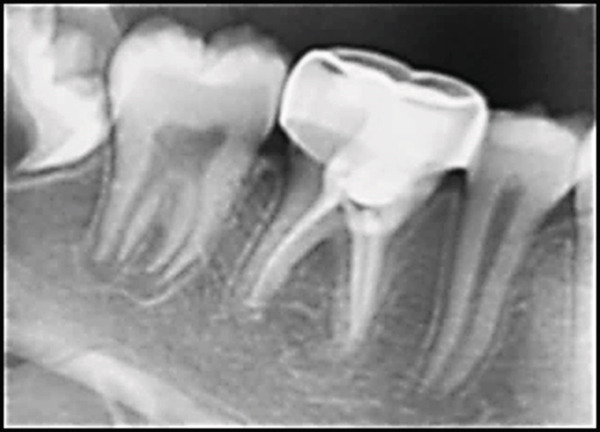
Postoperative radiograph of 46.

At the next visit, root canal treatment of the 46 could be completed with gutta‐percha because of her improved cooperative level. Coronal restoration of 46 was done, and a preformed SSC was placed at a subsequent visit (Figures 11 and 12).

**Figure 13:**
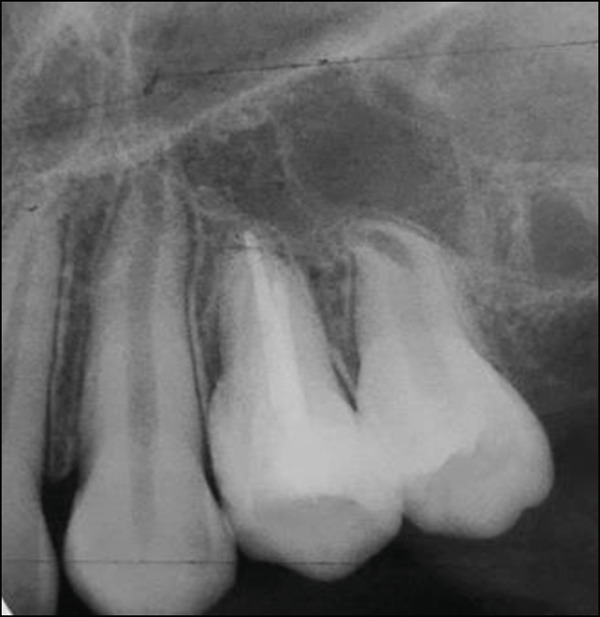
Postoperative periapical radiograph of 24.

At the following visit (Week 3), root canal treatment of 24 was concluded with gutta‐percha, and the coronal restoration was done using composite (Figure 13). CPP‐ACP product was prescribed for application over the teeth at night.

**Figure 14:**
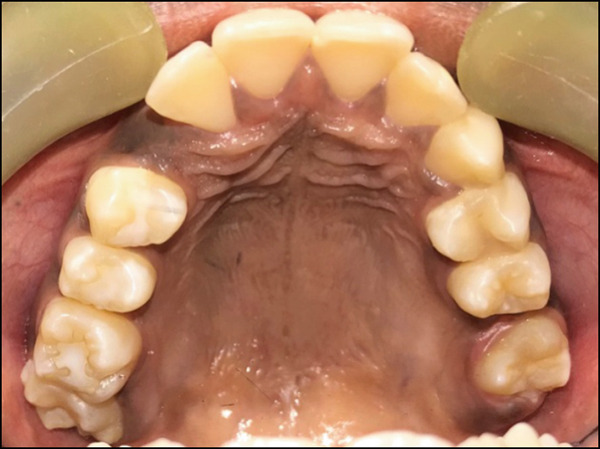
Postoperative upper arc.

**Figure 15:**
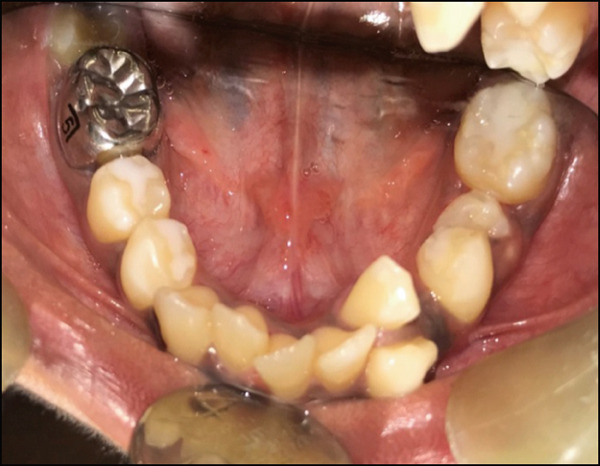
Postoperative lower arch.

**Figure 16:**
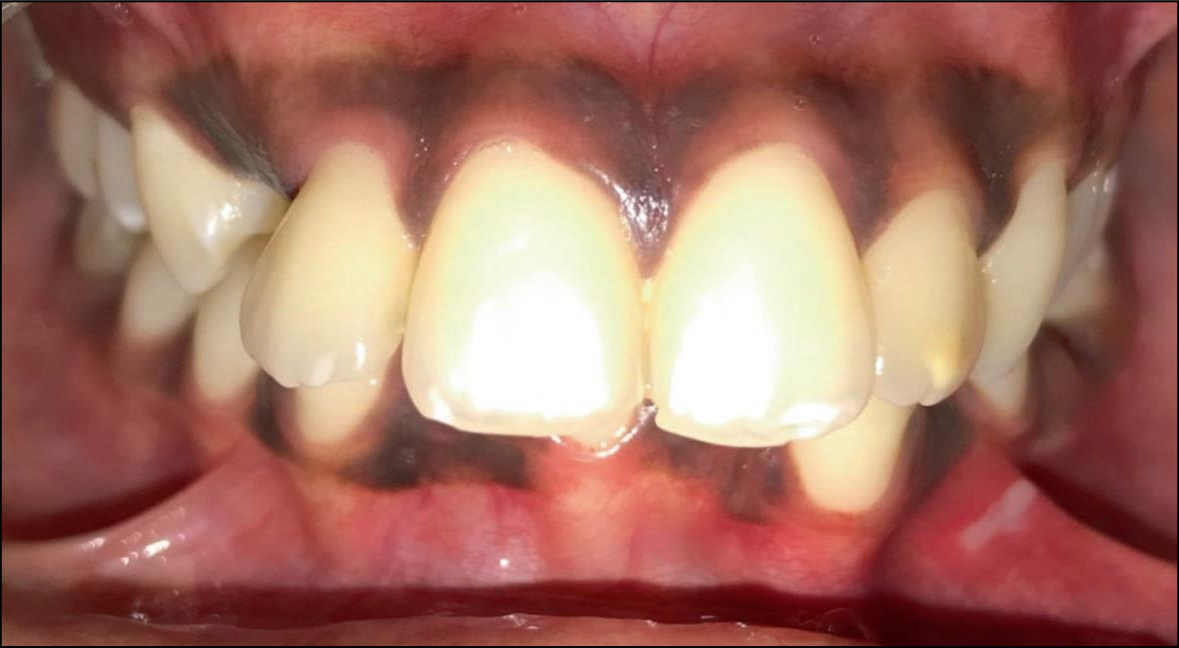
Postoperative labial view.

**Figure 17:**
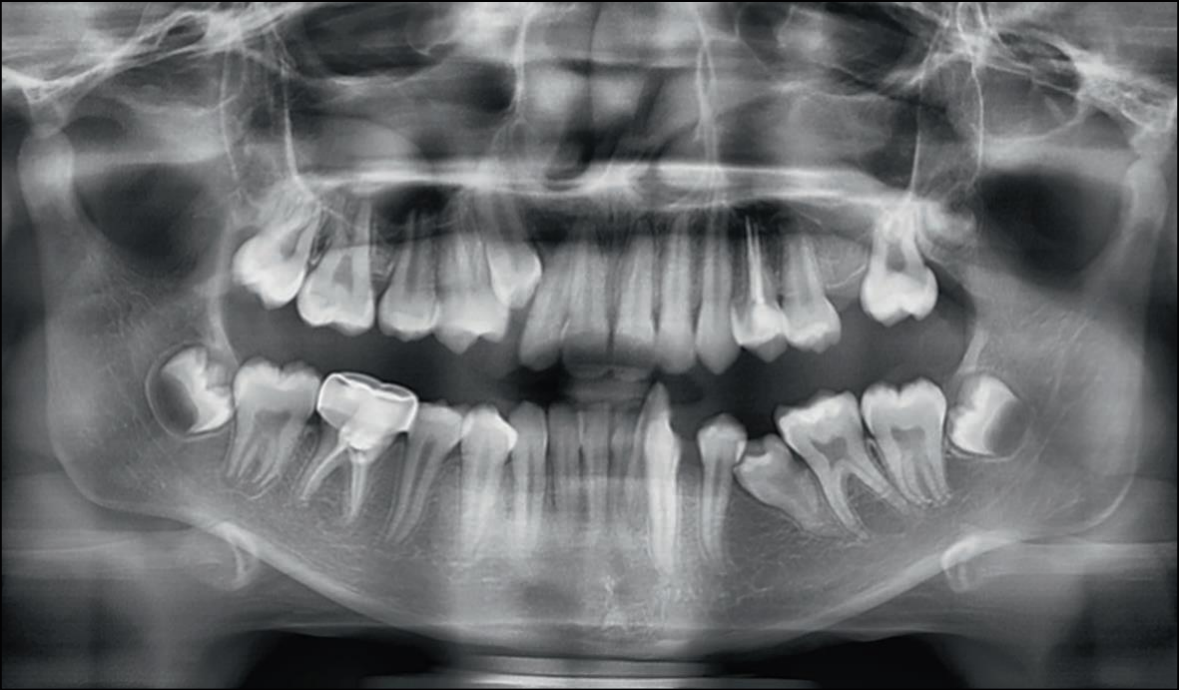
Postoperative DPT.

**Figure 18:**
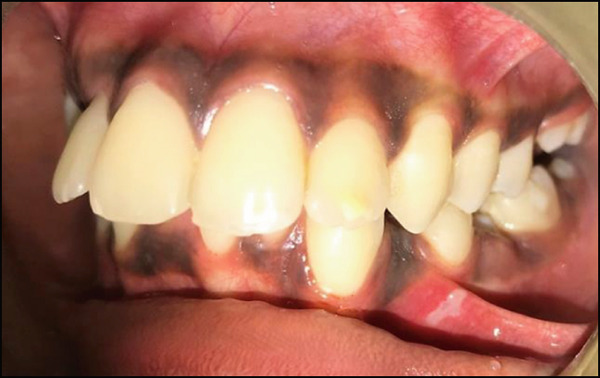
Postoperative left buccal view.

At the fourth week, overretained deciduous teeth (53, 55, 73 and 75) were extracted under local anaesthesia (Figures 14, 15, 16 and 17). A traumatic ulcer was noted on the labial mucosa in relation to the 33, 34 and 35 regions. It was observed during the review visits, and healing was evident without any recurrence (Figures 16 and 18).

Tooth 26 was extensively carious with pulpal involvement, requiring root canal therapy followed by indirect crown restoration. However, given its questionable long‐term prognosis and the associated financial burden of complex restorative care, extraction under local anaesthesia was considered the more appropriate option. As Tooth 27 had not yet erupted, removal of 26 provided the opportunity for 27 to erupt into the space, thereby supporting a more favourable long‐term outcome. This decision was made in consultation with the orthodontist, ensuring consistency with the overall treatment plan.

Preventive resin restorations of 16 and 36 were done, and resin‐based fissure sealants were applied to premolars.

**Figure 19:**
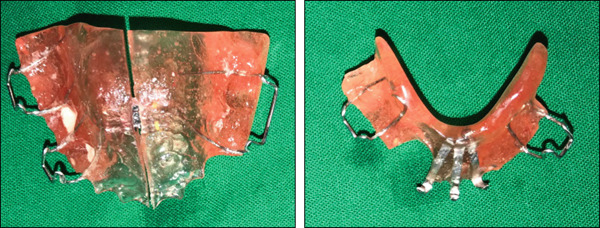
Twin Block appliance.

**Figure 20:**
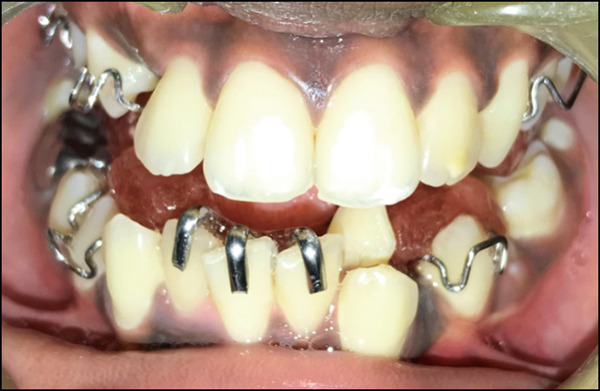
Front view with the Twin Block appliance.

**Figure 21:**
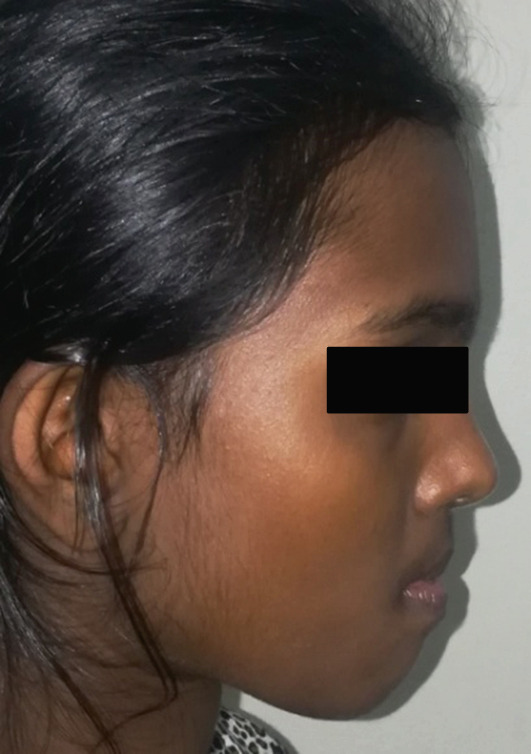
Lateral facial view with the appliance.

The patient and parents were highly motivated during the latter part of the management, and the child was cooperative. Orthodontic management was commenced after 3 months with the Twin Block functional removable appliance for the correction of Class II Division I malocclusion (Figures 19, 20 and 21). Interceptive management of buccally impacted 13 was carried out by extracting 53 since it was having an average prognosis for realignment [13, 14].

The patient was kept on regular maintenance care with 3‐month recall intervals. At review appointments, oral prophylaxis and reinforcement of oral hygiene measures with fluoride varnish applications were done. The eruptions of 27, 13 and 35 were observed, and when asked, the patient did not report any aesthetic concerns or hypersensitivity of the hypomineralised patches on incisors.

## 3. Discussion

The differential diagnosis of MIH includes fluorosis, enamel hypoplasia, amelogenesis imperfecta, white‐spot lesions and traumatic hypomineralisation. Distinguishing features that support a diagnosis of MIH are its typically asymmetrical presentation, well‐demarcated whitish to creamy yellow‐brown opacities with irregular margins, posteruptive enamel breakdown and the presence of atypical caries or restorations in otherwise low‐risk sites [4]. Severely affected molars carry up to a 10‐fold increased risk of caries compared with unaffected molars, and because MIH is common, it contributes substantially to the overall burden of childhood caries [5].

Hypersensitivity in hypomineralised enamel is a key driver of distress and poor cooperation. The porous enamel transmits thermal stimuli to the pulp, fostering chronic pulpal stress; pH changes in periapical tissues lower the activation threshold of pulpal nerves, so less stimulus is needed to evoke pain [10]. In practice, this hypersensitivity undermines toothbrushing, moisture control and anaesthesia, compounding disease progression.

Short‐term desensitisation can markedly improve cooperation. Applying fluoride varnish and placing interim GIC dressings 1–2 weeks before definitive care helps reduce hypersensitivity and allows subsequent procedures to be performed more comfortably [15]. In this case, varnish and GIC coverage over hypomineralised areas were used to good effect before definitive restorations. During endodontic procedures, adjunctive intraligamentary and intraosseous injections were employed under a rubber dam to improve anaesthetic success in MIH molars. To further mitigate sensitivity, saliva ejectors were used in preference to high‐volume suction, consistent with reports that high‐flow aspiration can exacerbate discomfort [16]. Inhalation sedation is often recommended to increase pain thresholds and cooperation in anxious children [12], but resource limitations precluded its use here.

Remineralising adjuncts can support sensitivity control and lesion arrest. CPP‐ACP products such as Tooth Mousse (10% CPP‐ACP) and MI Paste Plus (10% CPP‐ACP with 0.2% NaF, 900 ppm F) have demonstrated additive benefits when combined with fluoride compared with either agent alone [17, 18]. Other options include enamelon treatment gel (ACP with 970 ppm F) and NovaMin (bioactive glass ~18 *μ*m) which can occlude dentinal tubules and promote surface remineralisation, sometimes outperforming CPP‐ACP for surface effects [19]. In this patient, availability constrained the choice to Tooth Mousse, which was prescribed for nightly application.

Preventive fissure sealing should be tailored to eruption status and enamel quality. Where there is no posteruptive breakdown, resin‐based sealants are preferred; applying an adhesive beforehand can improve retention [20]. Because the protein‐rich surface of MIH enamel impairs bonding, deproteinising with 5% sodium hypochlorite or papain gel has been suggested to enhance bond strength [21]. In partially erupted, hypersensitive molars, interim sealing with high‐viscosity GIC is reasonable until isolation permits resin placement [4]. In this case, occlusal caries in 16 and 36 were restored with posterior resin composites after caries removal, and sound fissures were sealed with resin‐based materials following adhesive application; all premolars were sealed given the high caries risk.

Teeth with extensive defects, particularly with cusp involvement, are best managed with preformed metal/SSCs. SSCs are cost‐effective, require minimal tooth preparation, can be completed in a single visit, restore correct occlusal and interproximal contacts, eliminate hypersensitivity and protect compromised tooth tissue from further breakdown [9]. In this patient, 46 underwent root canal treatment and had lost substantial coronal structure, including the mesiobuccal cusp; a preformed SSC was therefore placed and cemented with luting GIC to provide durable full coverage.

Strategic extraction of compromised FPMs requires careful timing to optimise spontaneous mesialisation of second permanent molars (SPMs). When extracted at the ideal developmental window, the probability of ideal SPM positioning is approximately 94% in the maxilla and 66% in the mandible [22]. In the upper arch, complete space closure is common regardless of timing, and unerupted SPMs usually attain good occlusal positions after FPM removal [23]. In Class II cases, upper FPM extraction must be coordinated with incisor correction; where feasible, temporising the FPM until SPM eruption can preserve options for using the FPM space during fixed appliance therapy [23]. In this case, 26 was symptomatic with poor restorability and was extracted to allow 27 to erupt into position; Class II correction was planned with a functional appliance, with further orthodontic needs to be reviewed during maintenance.

Aesthetic management of hypomineralised incisors in young patients should be conservative due to large pulp sizes and ongoing eruption. Deferring definitive aesthetics is often appropriate, as opacities may diminish with time [24]. Conservative options include microabrasion, bleaching, etch‐bleach‐seal and resin infiltration. Microabrasion alone is less effective for MIH because the defect typically originates near the amelodentinal junction rather than at the surface [24], though combining microabrasion with CPP‐ACP has shown improved remineralisation [24]. Bleaching can help camouflage white opacities in adolescents [25], while the etch‐bleach‐seal technique targets yellow–brown staining [26]. For larger defects with exposed dentine, direct composite veneers may be used but require ongoing maintenance due to wear, staining and marginal chipping [9]. Porcelain veneers are generally reserved for patients over 18 years when other modalities are insufficient [9]. In this case, neither the patient nor her parents expressed aesthetic concerns, and the opacities were clearly visible only on Teeth 21 and 22. As the patient was 11 years old and the incisors had relatively large pulp chambers, any restorative intervention carried a higher risk of pulpal involvement. Therefore, the maxillary incisors were monitored without intervention.

Assessment of unerupted canines includes careful palpation for a buccal canine bulge from around age 8, inspection of lateral incisor inclination and radiographic localisation. Asymmetry in eruption timing between sides warrants imaging [27]. Localisation can be achieved with horizontal parallax (two periapicals or periapical plus anterior occlusal), which shows higher sensitivity for palatal impactions (88%) than vertical parallax with DPT plus occlusal (69%) [28]. Cone‐beam CT offers superior localisation and detects root resorption with approximately 50% greater sensitivity [29]. In this case, CBCT confirmed a buccally impacted 13 without evidence of adjacent root resorption. Using the prognostic factors of [13, 14] horizontal overlap, angulation to the midline, vertical crown height and apex position, the overall prognosis for realignment was judged average. Given that 53 was grossly carious with no sound coronal structure, interceptive extraction of 53 was undertaken, with planned monitoring of 13’s eruption over 12 months. Interceptive extraction of the deciduous canine can lead to complete resolution in around 62% of cases and favourable positional improvement in a further 17% [28]; palatally ectopic canines show path normalisation within 12 months in approximately 78% of cases [29].

Finally, the Class II Division 1 malocclusion was managed with a Twin Block functional appliance. Functional appliances are most effective when timed to coincide with the pubertal growth spurt [30]. Twin Blocks are relatively slim, permit freer mandibular movement and have minimal speech impact, which can improve compliance [31]. Expected effects are largely dentoalveolar: retroclination of upper incisors and distalisation of upper molars, with proclination of lower incisors and mesial movement of lower molars [32]. The patient was reviewed regularly to reinforce oral hygiene, monitor for new lesions and assess orthodontic progress. In similar cases, it would be interesting in the future to test potential effects of adjunctive treatments such as the use of biomimetic hydroxyapatite [33] or casein for long‐term prevention purposes [34].

In terms of medium‐ to long‐term management, Tooth 24, which underwent root canal treatment, will require definitive full coverage restoration to ensure durability, function and aesthetics. Additionally, given the severe lower anterior crowding and impacted 35, fixed appliance therapy is anticipated as part of the orthodontic phase of treatment. These interventions highlight the multidisciplinary nature of care in such complex cases. The patient remains under ongoing follow‐up, and future treatment phases will be planned according to clinical progress and growth considerations.

## 4. Conclusion

Early diagnosis of MIH allows more conservative and predictable management, whereas delayed identification often results in more complex treatment needs. Multidisciplinary management is essential for achieving successful outcomes in patients with complex restorative, behavioural and orthodontic needs, as illustrated in this case.

## 5. Limitations

### 5.1. Follow‐Up Duration

The follow‐up period reported was relatively short. A longer follow‐up would have provided more comprehensive evidence of both dentoalveolar and skeletal changes achieved with the Twin Block appliance. The patient, however, is still under ongoing follow‐up care, and extended monitoring is expected to yield further insights.

## Author Contributions

K.M.C.P. was responsible for the treatment of the patient and writing the case report. C.G.H. supervised the treatment and reviewed the manuscript. H.S.K.R. provided the orthodontic treatment.

## Funding

No funding was received for this manuscript.

## Disclosure

No individuals or third‐party services not listed as authors and not acknowledged elsewhere in the manuscript were involved in the research or the preparation of the manuscript. All authors have read and approved the final manuscript and agreed to be accountable for all aspects of the work. The authors confirm that this manuscript is original, has not been published elsewhere and is not under consideration by any other publication.

## Consent

Written informed consent for publication of the case details and accompanying images was obtained from the patient and her legal guardians. Additionally, written approval and informed consent to publish were obtained from the parents of the patient prior to submission of this manuscript.

## Conflicts of Interest

The authors declare no conflicts of interest.

## Data Availability

The authors confirm that the data supporting the findings of this study are available within the article.
